# Climate Change Impacts on Agricultural Production and Crop Disaster Area in China

**DOI:** 10.3390/ijerph17134792

**Published:** 2020-07-03

**Authors:** Zhen Shi, Huinan Huang, Yingju Wu, Yung-Ho Chiu, Shijiong Qin

**Affiliations:** 1 Business School, Hohai University, Changzhou 213022, China; 20051726@hhu.edu.cn (Z.S.); 1763510216@hhu.edu.cn (H.H.); 1863510326@hhu.edu.cn (Y.W.); 1863310220@hhu.edu.cn (S.Q.); 2 Department of Economics, Soochow University, 56, Kueiyang St., Sec. 1, Taipei 10048, Taiwan

**Keywords:** super undesirable dynamic SBM, agricultural production efficiency, spatial effect, Dagum Gini coefficient, kernel density

## Abstract

As one of the largest agricultural countries in the world, China has always paid close attention to the sustainable development of agricultural production efficiency. However, with global climate change, extreme weather has become an exogenous factor that cannot be ignored, as it affects agricultural production. Most of the existing studies only consider the domestic natural resources and economic factors, without fully considering the external climate factors. This paper uses the super undesirable dynamic Slacks-Based Measures (SBM) under an exogenous variable model to simulate the external environmental factors by adding extreme weather days. The Dagum Gini coefficient and kernel density estimation are used to explore the regional differences in agricultural production in China. The results show that the agricultural production efficiency is higher in the eastern region, and the difference in agricultural production efficiency among the provinces in the middle and western regions is large, showing a trend of polarization. The difference in the Gini coefficient between the middle and western regions is more significant. The main contribution factor of the Dagum Gini coefficient is the inter-regional difference. The regional concentration degree of agriculture in China is decreasing, the regional distribution of agricultural water resources is more balanced, and the national regional difference gradually decreases. Finally, some suggestions are put forward, such as extreme weather control, agricultural water supply, and water-saving measures.

## 1. Introduction

The efficiency of agricultural production is related to the total output value of agriculture and the foundation of national economic development. However, there is a complex standard to measure the efficiency of agricultural production. Therefore, the efficiency of agricultural production cannot be judged simply on the basis of the output value, nor only on the basis of how much production input can be measured. For this reason, the academic community has launched many significant explorations and produced highly effective research results. Based on the different boundary conditions from the perspective of research, scholars consider the efficiency of agricultural production in different regions from the perspective of time and spatial differences, climate disasters on crop production intervention, and other aspects. In this process, scholars have also explored many evaluation methods for agricultural production efficiency, including the Data Envelopment Analysis (DEA) model; the Slacks-Based Measures (SBM) model under undesirable output, derived from the DEA model; and the production function model. Some scholars also discussed the spatial differences in agricultural production, and have explored the balanced and high-quality development of agriculture by analyzing the differences in agricultural efficiency between different regions.

Among them, the lack of water resources has always been one of the important limiting factors for the sustainable development of agriculture in China. China is a large agricultural country, with agricultural water consumption accounting for more than 60% of the total water consumption, and irrigation water consumption accounting for 90% of the total agricultural water consumption. However, there is an uneven space–time distribution of water resources in our country and an unbalanced supply and demand between regions. The sdudy of water resources management also existed in agricultural irrigation, but there is still an unreasonable lack of water conservancy facilities and management mechanisms, which is a serious obstacle to the improvement of agricultural production efficiency. Therefore, this paper focuses on the agricultural production efficiency and put forward relevant solutions.

In the next part, we summarize the relevant literature published on the spatial differences in agricultural production efficiency and the impact of climate conditions on agricultural production efficiency, and describe the issue of agricultural production efficiency in China.

### 1.1. Literature Review

Based on the review of the previous literature, this paper summarizes the current literature in different aspects, as follows:

#### 1.1.1. Research on the Effect of Climate on Agricultural Production

Elias et al. [1] studied the changes in agricultural production caused by extreme temperature in the southwest of the United States, and described the agricultural pressure and adaptive response of the United States by analyzing the changes in variable elements. The results showed that the water shortage in the semi-arid areas of the southwest of the United States was becoming more and more serious, resulting in the reduction of crop production. Olen et al. [2] assessed the impact of water scarcity and climate on agricultural crop producers’ irrigation decisions in the United States. The results showed that the lack of water and extreme weather have a significant impact on the irrigation decision-making of producers. The use of sprinkler irrigation technology or extra water by producers should be used to reduce the risk of damage to crops caused by extreme weather, and then increase water consumption. Markovic et al. [3] studied the efficiency of irrigation scheduling for maize production in Croatia and found that the main factors affecting the irrigation efficiency under extreme weather conditions were determining the optimum water level for the soil water sensor and the relationship between the water table and root depth. Eggen et al. [4] studied the development of sorghum crop models under El Niño. The results showed that the incidence of sub seasonal failures in precipitation increased in the early rainy season, which had a side effect on sorghum yield. Olesen and Bindi [5] studied the impact of global warming on agricultural production in Europe, and the results showed that in the south, adverse factors will dominate, including water shortages and the possibility of increasing extreme weather events, which will lead to reduced harvests and a reduction in suitable agricultural planting area. Mishra et al. [6] studied the climate sensitivity of agricultural production in the state of Odisha, on the east coast of India. By studying the temperature and rainfall in the process of agricultural production, the Ricardo method was used to evaluate the impact of climate change on the net income of the agricultural production of Odisha. Wang et al. [7] selected the trend yield of each crop, and then constructed and calculated the probability density function curve and distribution function of the relative meteorological yield.

The probabilities of different production decline intervals have been estimated. Alboghdady and El-Hendawy [8] used panel data from 20 countries in the Middle East and North Africa from 1961 to 2009 to assess the impact of climate change and variability on agricultural production. The results showed that the increase in temperature in winter is 1%, which leads to the decrease of agricultural production by 1.12%. Olayide et al. [9] investigated the different effects of rainfall and irrigation on agricultural production in Nigeria, which provided reference for climate-smart agriculture (CSA) in Nigeria. Alboghdady and El-Hendawy [8] used a production function model and fixed effect regression analysis to evaluate and analyze the impact of climate change on variable agricultural production. The results showed that the increase in temperature in the winter was 1%, which led to a 1.12% reduction in agricultural production.

Barrios et al. [10] discussed the impact of climate change on total agricultural production in sub-Saharan Africa (SSA) and non-sub-Saharan Africa (NSSA). The results showed that climate change, measured by rainfall and temperature changes in the country, had become a major determinant of agricultural production in South Sudan. On the basis of the original Cobb–Douglas (C-D) production function, Kaimakamis et al. [11] built a new economic climate model by adding climate factors, and made an empirical analysis of the impact of climate change on food production, explaining the regional differences. Mardero et al. [12] utilized climate trend analysis and generalized additive models (gams) to analyze precipitation and temperature data from 1980 to 2010, proving the relationship between yield and climate variability. In order to clarify the temporal and spatial distribution of climate disasters and the response of wheat yield to disasters in the past 30 years, Shi and Tao [13] defined and calculated disaster indexes, such as the impact of climate disasters, the sensitivity of climate disasters, and the response index of wheat yield loss to the climate disasters. Based on the statistical data of agricultural disasters in the Heilongjiang Province from 1983 to 2013, Xing et al. [14] analyzed the occurrence area and change characteristics of different types of disasters. Finally, the degree of agricultural loss caused by these disasters was analyzed by a fuzzy comprehensive evaluation method. Xie et al. [15] used information diffusion technology and an information matrix to determine the distribution of drought risk in China’s main grain producing areas, and quantitatively analyzed the relationship between the annual drought rate and grain production loss. Xu et al. [16] used the production function model to demonstrate the quantitative relationship between the disaster area and the final yield of grain from the input factors of agricultural production and the disaster resistance ability. Zhang [17] used the methods of crop yield–climate analysis and regression analysis to analyze and quantify the relationship between corn yield fluctuation and agrometeorological disasters. Lesk et al. [18] estimated the global loss of grain production caused by extreme weather disasters reported during the period 1964–2007. The results showed that drought and extreme high temperature reduced grain yield by 9–10%. 

#### 1.1.2. Study on the Spatial Difference of Agricultural Production

Han and Wu [19] explored the impact of changes in China’s agricultural structure on factors such as energy intensity of agricultural production (EIAP). The results showed that the results of six vegetable production regions show great regional heterogeneity, which is mainly due to the scale economy effect and incremental effect of vegetable mechanization. On the basis of analyzing the heterogeneity of agricultural technology, Fei and Lin [20] used meta-frontier DEA to measure the agricultural energy efficiency. The results showed that the energy efficiency of the eastern region of China was significantly higher than that of the western region. Based on the provincial panel data of 1995–2014, Diao et al. [21] analyzed the agricultural productivity and its regional differences in China. The results showed that TFP growth in the central and western regions was much higher than that in the eastern regions. In 2014, the most effective decision-making unit was the western region. Ito [22] measured the regional differences of agricultural productivity in China, and then tested the validity of the hypothesis related to agricultural technology. Zhang et al. [23] compared the agricultural disasters in the north and south of China, and the results showed that the losses in the north increased by about 0.6% every ten years, close to twice that in the south of China. In addition, agriculture in northern China was more sensitive to precipitation change, while agriculture in southern China was more sensitive to temperature change. Wagan et al. [24] compared the agricultural production efficiency of China and Pakistan, and the results showed that the overall efficiency of China’s agricultural production was higher than that of Pakistan. Although Pakistan’s agricultural production had increased, China’s agricultural production had higher efficiency because of its strong dependence on technology; Pakistan needed to apply new agricultural technology. Based on the panel data of the Songnen Plain in the Heilongjiang Province, Yang et al. [25] used quantitative and spatial analysis methods to explore the problem of agricultural production efficiency in this area. The results showed that agricultural production showed a growth trend from “high in the southwest and low in the northeast” to “high in the middle and low in the surrounding areas”, with obvious regional differences.

According to the agricultural production data of 31 provinces in 2014, Li et al. [26] used the DEA method to evaluate the comprehensive efficiency of agricultural production investment in China. The results showed that there was a big gap among the eastern, central, and western regions. Xue et al. [27] used the econometric model to classify the agricultural water environment efficiency of China in 2013, and analyzed the spatial effect and influencing factors of the agricultural water environmental efficiency of China using a spatial econometric model. The results showed that the spatial distribution of the agricultural water environmental efficiency is uneven, showing a gradual decrease from east to west. Li et al. [28] studied the theoretical and practical productivity of farmland in Zhejiang. The results show that the productivity in the north of the middle plain is the highest, while that in the southwest mountainous area is the lowest. Zhang and Zhu [29] evaluated the efficiency of agricultural water use in the Heilongjiang province, and guided the scientific water-use strategy of Heilongjiang Province according to the results. Sun et al. [30] studied the agricultural water footprint in the Hetao irrigation area, and the results showed that the water footprint of five counties in the Hetao irrigation area was significantly different. Neumann et al. [31] used econometric methods and spatial analysis to explore the maximum yield, yield gap, and the efficiency of wheat and other crops. The results show that the actual grain output in some areas is close to its maximum, while there is still a large gap in other areas. Crain et al. [32] studied the spatial variability of agricultural crops, and the results showed that significant differences in winter wheat yield were found in the adjacent 1 m × 1 m plot.

In order to study the actual agricultural production process more scientifically, this paper creatively adds extreme weather as exogenous variables to build a super undesirable dynamic SBM model. Based on the eastern, middle, and western regions of China, this paper also uses the kernel density estimation method and Dagum Gini coefficient to explore the dynamic evolution law of agricultural production efficiency, as well as the regional differences.

### 1.2. Issue of Agricultural Production Efficiency in China 

In 2017, the opinions of the CPC Central Committee and the State Council on deepening the structural reform of agricultural supply side and accelerating the cultivation of new driving forces for agricultural and rural development were released. The document clearly points out that it is necessary to further promote the agricultural supply around the change of market demand, improve the quality and efficiency of the agricultural supply system, and promote the high-quality development of rural areas through the rational use of agricultural resources. It can be seen that improving the efficiency of agricultural production has become an important problem to be solved. The key link for improving agricultural production efficiency is to optimize agricultural water-use efficiency and improve agricultural productivity through effective input of agricultural water. The national agricultural water-saving program (2012–2020) issued by the general office of the state council in 2012 clearly emphasizes the use of comprehensive measures, including economic, administrative, legal, scientific, technological, and engineering measures, to promote the construction of agricultural water-saving systems with Chinese characteristics. Improving the efficiency of regional agricultural production is an important measure to save the scarce agricultural water resources, which requires the promotion and coordination of national policies. With the completion of the middle route and the eastern route of the south-to-north water diversion project, as well as the commissioning of the Three Gorges and Gezhou dams and other water conservancy facilities, the uneven distribution of water resources in China has been alleviated. However, the problem of regional agricultural production efficiency remains to be solved. Therefore, studying the trend of agricultural production efficiency over time and exploring the differences in agricultural production efficiency between regions can further explore the problems existing in agricultural production in various regions, so as to formulate relevant policies for regional governments and provide a relevant basis for the healthy development of agricultural production.

Agricultural production input and agricultural output constitute a relatively complex system. Extreme weather conditions as external factors have a great impact on agricultural production. China is one of the countries with the most severe climatic disasters. Due to the wide latitude in the north and south of China’s territory, all regions are faced with extreme weather, including drought, frost, flooding, and other disasters. In 2018, 19,260 hectares of crops were affected, posing a serious threat to China’s agricultural water and production security. Under extremely high temperature weather, land surface moisture evaporates quickly, reducing soil moisture and thus badly affecting the growth of the crops. Extreme weather conditions of low temperatures, and especially when co-occurring with continuous rainfall, may reduce agricultural water; however, the cold frost can lead to winter crop output, of which the winter wheat in the north is the main representative crop. Thus, the cold weather there has an important intervention effect on the wheat growth cycle, which will affect the whole crop production schedule for farming. Therefore, the crop output will be highly affected by future warmer temperatures. Therefore, as an external influence variable, the number of extreme weather events has an intervention effect on the water-use efficiency (WUE) of crops. On the premise of ensuring agricultural production efficiency, China should consider the impact of climate disasters on agricultural production. Therefore, based on the existing extreme weather disasters in China, it is of great practical significance to explore the issue of agricultural production efficiency in China.

Through studying the relevant literature on agricultural production efficiency, most scholars have started their discussion from river basins or grass-roots agricultural water facilities in China, while few studies have considered the agricultural production efficiency in China under extreme weather conditions. Unlike industry and some services, the impact of climate on agriculture should be taken into account in its input–output models. At present, existing research on agricultural production efficiency mainly include regional agricultural production efficiency or agricultural water-saving measures, and their research and analysis methods mainly include the DEA model, the empirical analysis method, and the stochastic frontier production function model. Since Gini [33] put forward the Gini coefficient, it is widely used to investigate the degree of regional income difference. Therefore, many scholars have carried out calculations of the Gini coefficient to explore the degree of agricultural regional differences given various factors.

In this context, this paper uses the SBM model to explore and analyze 30 provinces and regions in China. Then, the Dagum Gini coefficient calculation and kernel density estimation analysis method are used to explore the dynamic evolution law of agricultural production efficiency and regional differences in China. Furthermore, according to the change in regional differences, the paper puts forward relevant solutions to make a certain contribution to the efficiency of agricultural production in China. The innovation of this paper is as follows: (1) From a national level, we explore China’s agricultural production efficiency, through the selection of those input and output variables related to helping explore the defects and problems of agricultural production in China, from the macro-level controls of China’s agricultural production; (2) the Dagum Gini coefficient is used to calculate the differences between the eastern, middle, and western China regions. The evolution of the Dagum Gini coefficient from 2010 to 2017 is analyzed and the important contribution objects of the Dagum Gini coefficient is analyzed and explored in connection with the differences in agricultural production in China; (3) the concentration degree of agricultural production efficiency in different regions of China and the evolution of their dynamic differences are explored by using the kernel density estimation method; and (4) based on the SBM model, extreme weather days in various regions are added as external variables into the model analysis, which makes the model closer to the actual situation of agricultural production.

## 2. Methods

### 2.1. Super Undesirable Dynamic SBM Under an Exogenous Model

Compared with the traditional ratio method and absolute efficiency analysis method, Data Envelopment Analysis (DEA) is a non-parametric, technical efficiency analysis method, based on the relative comparison between evaluation objects. It was first proposed by Charnes et al. [34] in the United States. DEA has many advantages. Firstly, it can deal with the problem of multi input and multi output without building a production function to construct the parameters. Secondly, it can evaluate the efficiency of the comprehensive index of the studied elements and describe the production status of all elements. Thirdly, it is not affected by human subjective factors. The required weight is generated by mathematical programming and does not need to be given a weight in advance. Fourthly, the DEA model is not affected by the dimension of the input–output index. Finally, the DEA model can put forward an improvement direction for the inefficient decision-making units (DMUs) through the analysis of slack variables. Therefore, for this study we chose the “super undesirable dynamic SBM model under an exogenous variable”, one of the latest DEA models, as the research method.

When evaluating the efficiency of each evaluation unit, sometimes the efficiency value of multiple evaluation units is 1, especially when there are many input and output indicators, meaning the number of effective DMUs will increase, which leads to the problem of insufficient judgment in the DEA. Andersen and Petersen [35] first proposed a method to further distinguish the effective degree of the DMU, which solved the problem that the efficiency value of the evaluation unit was too large. This new model was called the Super DEA model. In this model, the evaluated decision-making units (DMUs) are removed from the reference set; that is to say, the efficiency of the evaluated DMU is obtained by referring to the frontier of other DMUs, and the efficiency value of the effective DMU is generally greater than 1, so the effective DMU can be distinguished. By evaluating the efficiency value of one unit in the selected set separately, on the basis of the remaining evaluation unit, the efficiency of the evaluation unit is recalculated, and the efficiency boundary of the evaluation unit is sorted, so that the excess of the rejection efficiency value (efficiency value) may be greater than or equal to 1.

In the radial DEA model, the measurement of the degree of inefficiency only includes the proportion of increase and decrease of all inputs and outputs. For the invalid DMU, the gap between the current state and the effective target value includes not only the parts of equal proportion improvement, but also the parts of relaxation improvement. However, the part of relaxation improvement is not reflected in the calculation of the efficiency value. Therefore, Tone [36] first proposed an efficiency value estimation model based on margin variables. This model adopts the non-radial estimation method and considers slacks of input and output at the same time. The estimated efficiency value is between 0 and 1, which is called the SBM model. However, under this model, the same SBM efficiency value of multiple decision-making units would still be 1, so Tone [37] proposed a slashed-based measure of a super-efficiency model.

Klopp [38] first proposed window analysis for dynamic analysis, followed by the Malmquist index of Färe et al. [39], divided into catch up and innovation effects; however, these analyses did not analyze the influence of “the effect of carry-over activities” in these two periods, while Färe and Grosskopf [40] first put inter-connecting activities into the dynamic. After Färe et al., Tone and Tsutsui [41] extended the model to the dynamic analysis of a slacks-based measure. Due to Tone and Tsutsui’s dynamic DEA model not considering the undesirable, exogenous variables, the entire unit’s efficiency value with ‘1’ was too much in the results. Therefore, the dynamic DEA was combined with Tone’s [37] super SBM, as well as with that of Tone and Tsutsui [41]. 

Considering the exogenous variables, the super undesirable dynamic SBM model assesses the efficiency of agricultural water use, to avoid the efficiency value and improve the undervalued or overvalued spaces. Considering the exogenous variables, the following proposes the model structure of the super undesirable dynamic SBM model.

Suppose the observations make up a J (J = 1…n) dimension DMU set in which the DMU under evaluation is represented by DMU_0_ and is subject to DMU_0_∈J. The input and output used to compute the efficiency are labeled as m inputs X_ijt_ (I = 1…m) and s outputs Y_ijt_, respectively. Let output Y be divided into (Y^g^, Y^b^), where Y^g^ is a desirable output, Y^b^ is an undesirable output, and $Z^{\mathrm{input}}$ is carried over from period t to period t + 1. $E_{\mathrm{ajt}}\left(a=1\ldots u\right)$ is an exogenous variable that is outside of a given economic model. It often has an impact on the outcome of the model. The following is the non-oriented model:

(1) $$\theta_{0}^{*}=\min\frac{\frac{1}{T}\sum_{t=1}^{T}W^{t}\left[1-\frac{1}{m+ninput}\left[\sum_{i=1}^{m}\frac{s_{it}^{-}}{x_{iot}}+\sum_{r=1}^{nbad}\frac{s_{rt}^{input}}{z_{rot}^{input}}\right]\right]}{\frac{1}{T}\sum_{t=1}^{T}W^{t}\left[1+\frac{1}{s_{1}+s_{2}}\left[\sum_{l=1}^{s_{1}}\frac{s_{jt}^{+g}}{y_{lot}^{g}}+\sum_{l=1}^{s_{2}}\frac{s_{jt}^{-b}}{y_{lot}^{b}}\right]\right]}$$

Equation (1) is the connection equation between t and t + 1.

(2) $$\{\begin{array}{l} \sum_{j=1,\neq 0}^{n}z_{ijt}^{a}\lambda_{j}^{t}=\sum_{j=1,\neq 0}^{n}z_{jit}^{a}\lambda_{j}^{t+1}\;(\forall i;t=1,\ldots ,T-1)\\ x_{iot}=\sum_{j=1,\neq 0}^{n}x_{ljt}\lambda_{j}^{t}+s_{it}^{-}\;(i=1,\ldots ,m;t=1,\ldots ,T)\\ y_{lot}=\sum_{j=1,\neq 0}^{n}y_{ljt}^{+g}-s_{It}^{+g}\;(I=1,\ldots ,s1;t=1,\ldots ,T)\\ y_{lot}=\sum_{j=1,\neq 0}^{n}y_{ljt}^{-b}\lambda_{j}^{t}+s_{It}^{-b}\;(I=1,\ldots ,s2;t=1,\ldots ,T)\\ z_{rot}^{input}=\sum_{j=1,\neq 0}^{n}z_{rjt}^{input}\lambda_{j}^{t}+s_{rt}^{input}\;(r=1,\ldots ,ninput;t=1,\ldots ,T)\\ E_{aot}=\sum_{j=1,\neq 0}^{n}E_{ajt}\lambda_{j}^{t}\;\left(a=1,\ldots ,u;t=1,\ldots ,T\right)\\ \sum_{j=1,\neq 0}^{n}\lambda_{j}^{t}=1\;(t=1,\ldots ,T)\\ \lambda_{j}^{t}\geq 0,s_{it}^{-}\geq 0,s_{It}^{+}\geq 0,s_{It}^{-b}\geq 0,s_{rt}^{+g}\geq 0 \end{array}$$

The super-efficient solution is

(3) $$\rho_{0t}=\frac{1-\frac{1}{m+ninput}[\sum_{i=1}^{m}\frac{S_{it}^{-}}{x_{iot}}+\sum_{r=1}^{nbad}\frac{S_{rt}^{input}}{Z_{rot}^{input}}]}{1+\frac{1}{S1+S2}[\sum_{l=1}^{S1}\frac{S_{jt}^{+g}}{y_{lot}^{g}}+\sum_{l=1}^{S2}\frac{S_{jt}^{-b}}{y_{lot}^{b}}]}\;i=\left(1,\ldots ,T\right)$$

### 2.2. Dagum Gini Coefficient

Considering the subgroup sample distribution, Dagum [42] proposed a new method of Gini coefficient decomposition, which solved the overlapping between sample data and the source of regional overall difference. This paper uses the Dagum Gini coefficient and its subgroup decomposition method to study the climate’s impact on the water-use efficiency in agriculture in our country, to study any regional differences. According to the Gini coefficient and its subgroup decomposition method proposed by Dagum, China is divided into three regions, namely, k = 3, and j and h are, respectively, k regions (different regions in the eastern, middle, and western region; j = 1,2..., k; H = 1,2...K, and j ≠ h); n is the number of provinces (cities) in the country, *n* = 30; n is the agricultural production efficiency of province i (r) (city) in region j (h) under the influence of climatic factors; and y is the arithmetic average of the national agricultural production efficiency under the influence of climatic factors. The calculation formula of the Dagum Gini coefficient and its subgroup decomposition method is

(4) $$G=\frac{\sum_{j=1}^{k}\sum_{h=1}^{k}\sum_{i=1}^{n_{j}}\sum_{r=1}^{n_{h}}\left|y_{ji}-y_{hr}\right|}{2n^{2}\overset{-}{y}}$$

Dagum decomposed the overall Dagum Gini coefficient G into three parts: intra-regional difference contribution (G_w_), inter-regional net value difference contribution (G_nb_), and intensity of transvariation (G_t_), which met G = G_w_ + G_nb_ + G_t_.

### 2.3. Kernel Density Analysis

As a non-parametric estimation method, kernel density estimation is mainly used to obtain the distribution pattern of random variables by smoothing the probability density of the random variables based on the kernel function, which is widely used in the analysis of regional differences. X1, X2..., Xn is the sample of a unary continuous population, and Formula (5) is the kernel density estimation of the density function f(x) at any point x. Where f(x) is defined as the density function, K(·) is the kernel function, and h is the bandwidth.

(5) $$f_{h}(x)=\frac{1}{nh}\sum_{i=1}^{n}K(\frac{x-X_{i}}{h})$$

In this paper, the commonly used Gaussian kernel function was selected to estimate the kernel density curve of the distribution pattern of agricultural production efficiency under the influence of climatic factors in China. Based on the sample data, the dynamic evolution law of the distribution of agricultural production efficiency under the influence of climatic factors was described from the time dimension.

## 3. Results

### 3.1. Data Description

#### 3.1.1. Explanation of Variables

This paper takes 30 provincial administrative units in China as research objects, and analyzes the agricultural production efficiency of the research objects based on the one-stage dynamic super-efficiency SBM model. As the study’s focus is on the provinces of the Chinese mainland, Taiwan, Hong Kong, and the Macao special administrative regions were not analyzed. In addition, due to limited data of the Tibet autonomous region, it was also not included.

According to the seventh five-year plan of the fourth session of the sixth National People’s Congress, the Chinese mainland is divided into eastern, middle, and western regions. Inner Mongolia and Guangxi are classified as the western region because their per capita gross domestic product (GDP) is comparable to the average of the 10 provinces in the western region. See Table 1 for details.

Considering the availability of data, this paper analyzed the variables’ efficiency for 30 provinces in China from 2010 to 2017. In the first stage of the input process, agricultural water consumption, agricultural employees, cultivated irrigated area, and fixed assets were used as the input indicators, while gross output value of agriculture was taken as the desired output, and the disaster area for crops is taken as the undesired output. Extreme weather is taken as the exogenous variable of the model, and fixed assets is taken as the carry-over variable. See Table 2 for details.

Details of the specific variables are explained as follows:

① Agricultural water consumption (AWC). It refers to the sum of the irrigation water for farmland, forest fruit land, grassland, fish ponds, and livestock.

② Agricultural employees (AE). It refers to the total number of people who work in a primary industry and are paid for their labor in the first industry, including on-the-job worker, re-employment of the retired personnel, the foreign staff working in the various units, Hong Kong, Macao and Taiwan personnel, part-time staff, personnel from other units on loan, and the second unit outside professionals, etc., but excluding the employees who leave the primary industry but retain labor relations.

③ Cultivated irrigated area (CIA). It refers to the cultivated land area with a certain water source: a relatively flat plot with supporting irrigation projects or equipment, which can be irrigated normally in normal years. It is an important index reflecting the construction of farmland water conservancy in China.

④ Gross output value of agriculture (GOVA). It refers to the total value of all agricultural, forestry, animal husbandry, and fishery products, expressed in monetary terms and various supporting service activities for agricultural, forestry, animal husbandry, and fishery production activities, which reflects the total scale and total results of agricultural, forestry, animal husbandry, and fishery production in a certain period.

⑤ Crop disaster area (CDA). It refers to the sown areas affected by floods and drought, which results in a lower crop yield than in normal years. It includes the disaster area and dead area.

⑥ Extreme weather days (EWD). It refers to the total number of days with temperatures below 5 degrees Celsius and above 32 degrees Celsius in each province.

⑦ Fixed assets (FA). Is refers to the volume of construction and the acquisition of primary industry fixed assets in monetary terms. According to the depreciation of China’s fixed assets, the depreciation rate of physical capital is 0.096. The formula of permanent inventory method is as follows:

(6) $$K_{it}=K_{i,t-1}\left(1-\delta \right)+I_{it}$$

In the formula, *k_it_* and *k*_*i,t*−1_ are, respectively, the investment stock of this year and the investment stock of last year, and δ stands for the depreciation rate.

**Figure 1:**
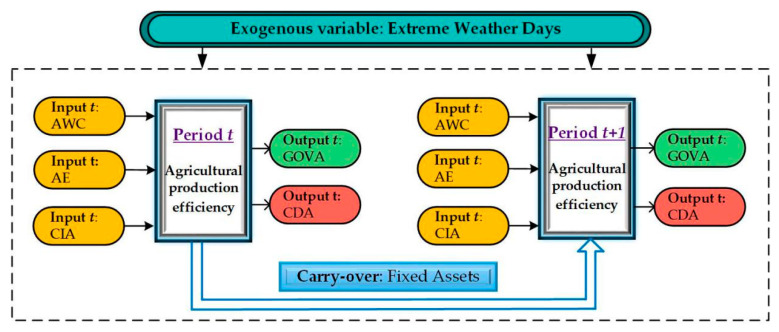
The Slacks-Based Measures (SBM) dynamic model.

Figure 1 is the process analysis diagram of the SBM dynamic model, which shows the process thinking of this paper by using flow chart. See Figure 1 for details.

#### 3.1.2. Data Description

In this paper, the agricultural input–output data of the whole country, the east, the middle, and the western regions from 2010 to 2017 were selected to measure the mean value, maximum value, minimum value, and standard deviation of agricultural water consumption, agricultural employees, cultivated irrigated area, fixed assets, gross output value of agriculture, crop disaster area, and extreme weather days. The data for agricultural water consumption, cultivated irrigated area, fixed assets, the gross output value of agriculture, and the crop disaster area were taken from China’s statistical yearbooks [43] from 2011 to 2018. The data regarding agricultural employees were from the local statistical yearbooks [44] of the provinces. The data of the extreme weather days were from online sources [45]. See Table 3 for details.

### 3.2. Agricultural Production Efficiency Analysis

The overall efficiency level of the eastern region is the best among the three regions, and the overall efficiency level of most provinces is greater than 1, including Shanghai, Shandong, Tianjin, Beijing, Jiangsu, and other provinces. Zhejiang has the lowest efficiency level, with the overall efficiency level of 0.857. The overall efficiency shows a downward trend, from 1 to 0.804 in the period of 2010–2017, but still at a high level of efficiency.

Among the middle regions, Henan and Heilongjiang have higher efficiency levels of 1.11 and 1.107, respectively; however, the overall efficiency level is around 0.5 in most provinces, such as Shanxi, Jilin, Anhui, and Jiangxi. The overall efficiency of Hubei and Hunan is around 0.7, but the overall efficiency level shows a downward trend, falling to 0.541 and 0.403, respectively, in 2017.

In the western region, its overall efficiency shows a trend of polarization. The efficiency level of Sichuan, Chongqing, Guizhou, and Shaanxi is above 1, while the efficiency level of Yunnan, Gansu, Ningxia, and Inner Mongolia is below 0.4. Among them, Ningxia has the lowest efficiency level, with the overall efficiency value fluctuating around 0.3 from 2010 to 2017.

It can be seen from the above situations that the efficiency levels of the three regions are different, and the internal differences between the western region and the middle region are also large. Table 4 summarizes the overall efficiency levels of the studied provinces in China from 2010 to 2017.

In the eastern region of China, Hebei, Beijing, Tianjin, Shandong, and Jiangsu have maintained high efficiency levels, indicating that the eastern route of China’s south-to-north water diversion project has played an important role in the eastern part of the country. As the Yellow River flows through Inner Mongolia, Ningxia, and Gansu, the agricultural production efficiency fluctuates between 0 and 0.4, which means the efficiency level is relatively low. In the middle region, the efficiencies of Hubei, Hunan, Jiangxi, and other provinces are relatively low. Jiangxi has the lowest agricultural production efficiency. It can be seen that there are still problems in agricultural production efficiency in the middle region where the Yangtze River flows.

**Figure 2:**
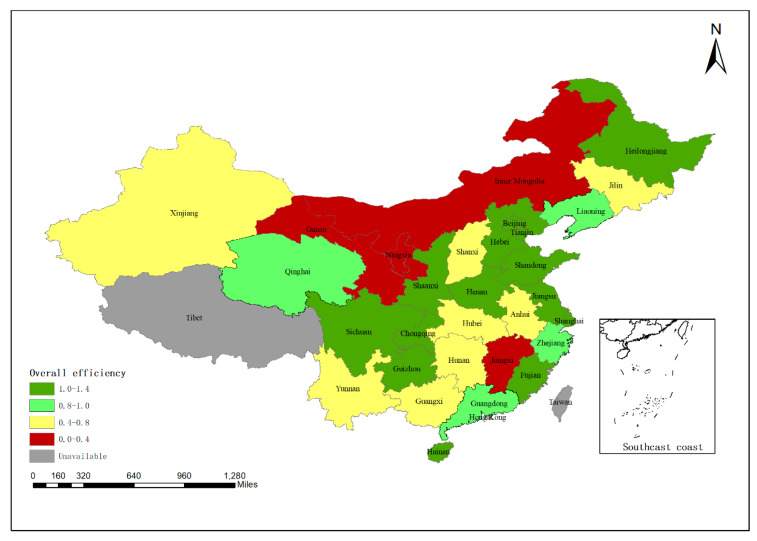
Geographical distribution of the overall agricultural production efficiency (China).

Figure 2 shows the level of agricultural production efficiency in the different regions of China by using the bitmap.

By calculating the efficiency values of the east, middle, west, and the whole nation in 2010–2017, the average efficiency values of each region show a downward trend. In 2010–2012, the national average efficiency dropped the most significantly, from 1.34 to 1.12. The rest of the year also showed a downward trend, falling to 0.97 in 2017. Among them, the average efficiency of the eastern region is the highest, and the middle region is the lowest. In 2015–2016, the efficiency of the eastern region shows a temporary upward trend, but in 2017 it dropped to about 1.

In 2010–2011, the average efficiency values in the middle and western regions were similar. However, in the following years, the efficiency level of the middle and western regions is higher than that of the middle region, and the gap between the two regions gradually widens to about 0.3 in 2017.

**Figure 3:**
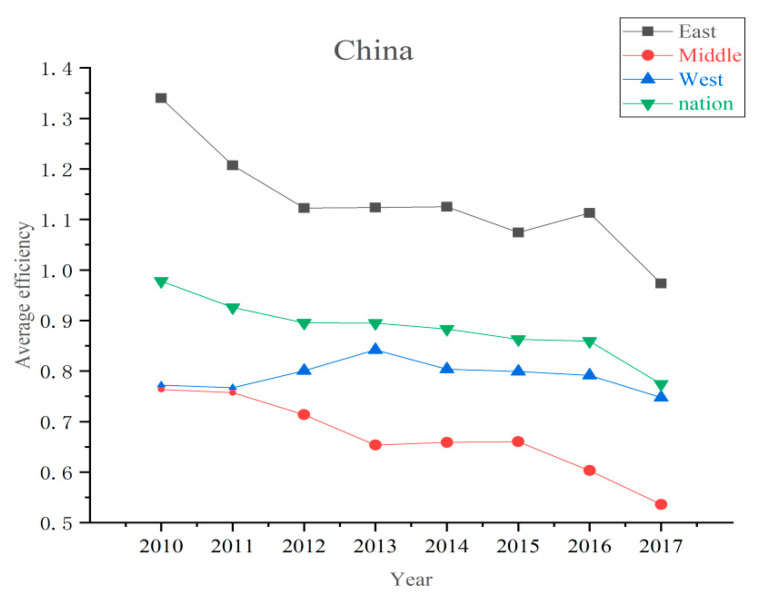
Average efficiency from 2013–2016 for the east, middle, west, and nationwide (China).

In general, all regions should strengthen the improvement of agricultural production efficiency and effectively curb the decline in agricultural production efficiency. Figure 3 shows the average efficiency of agricultural production in eastern, middle, and western China in 2010–2017.

### 3.3. Spatial Difference Analysis

In general, the overall Dagum Gini coefficient of China’s agricultural production efficiency fluctuates between 0.18 and 0.26, and shows a continuous decline from 0.2445 to 0.1810 during 2010–2012. Subsequently, it shows an upward trend, reaching a peak of 0.2604 in 2017. It can be seen that the overall regional differences in China show a U-shaped fluctuation within the research period, and the overall regional differences show a growing trend.

From the perspective of intra-regional differences, the differences in the eastern regions are the lowest, while the difference between the middle and western regions fluctuates around 0.2, showing a high level of intra-regional differences. In the eastern regions, the level of intra-regional differences generally shows a downward trend but shows a slight upward trend from 2014 to 2017, rising to 0.1254 in 2017. In the middle regions, the overall trend is that of a rise, from 0.2130 in 2010 to 0.2634 in 2017, a significant increase of 23.7%. The western regions show the most significant increase, from 0.2136 in 2010 to 0.2919 in 2017, an increase of 36.7%, indicating greater intra-regional differences.

From the perspective of the evolution trend among regions, the level of inter-regional differences in agricultural production efficiency in China tends to rise within the research scope, but the levels of the east–west regions tend to decline. In 2010, the level of the east–west and east–middle regions fluctuated around 0.28, while the level of the east–west regions was relatively low, only 0.217. From 2011 to 2017, the level of the east–west regions is significantly lower than that of the middle–east regions, while the level of the east–middle and middle–west regions shows an alternating rise, rising to more than 0.31 in 2017.

From the perspective of the evolution trend of the inter-regional differences’ sources and contribution rates, the contribution rate of the inter-regional differences was the highest from 2010 to 2017, with an average annual rate of over 46% in 8 years, and the overall fluctuation was relatively smooth. The contribution rate of intra-regional differences is second only to that of inter-regional differences, and the evolution is flat from 2010 to 2017, fluctuating at around 26%. The intensity of transvariation is the one with the lowest contribution rate. From 2010 to 2017, it fluctuated at around 20%. However, it showed an upward trend from 2015 to 2017 and increased to 25.77% in 2017. It can be seen that China’s inter-regional differences are the main source of China’s overall regional differences.

Table 5 summarizes the regional differences of China’s agricultural production, including the Dagum Gini coefficient within and between regions, as well as the contribution rate of the overall differences.

### 3.4. Kernel Density Estimation

On the whole, there are differences in the kernel density curves of the national, eastern, middle, and western regions. However, the peak value of each region gradually moves to the left, and the efficiency level tends to decline. The density distribution in the eastern region fluctuates greatly.

From 2010 to 2017, the peak value of the kernel density estimation curve showed a downward trend, indicating that the concentration degree of agricultural production efficiency shows a downward trend. From 2016 to 2017, the estimated curve shape of the national kernel density changes from a multi-peak shape to a single-peak shape, with the peak value decreasing and kurtosis widening. It shows that the differentiation degree of the whole country reduces.

The kernel density estimation curve of agricultural production efficiency in the eastern region moves slightly to the left, and the overall efficiency level shows a downward trend. From 2015 to 2017, the peak in the eastern region gradually increases, especially in 2017. It can be seen that the differentiation of the eastern region has increased. Moreover, in 2017, the peak in the eastern region increased significantly, and the concentration in the eastern region also increased.

In the middle region, the right tail of the kernel density estimation curve moves slightly to the left, and the level of agricultural production efficiency decreases. In addition, the kernel density estimation curve changed from a single-peak shape to a multi-peak shape, and the regional difference increased as well. In 2017, the peak density curve showed an obvious upward trend, with increasing concentration and polarization in the middle region.

From 2010 to 2013, the peak value of the kernel density estimation curve in the western region gradually decreased, and the regional concentration also decreased. From 2014 to 2017, the kernel density curve changes from multi-peak shape to single-peak shape, indicating that there is a “spillover effect” between provinces in the western region and the regional gap was narrowed. However, in 2017, the kernel density curve in the western region changed to a multi-peak shape again, and the regional gap further increased.

**Figure 4:**
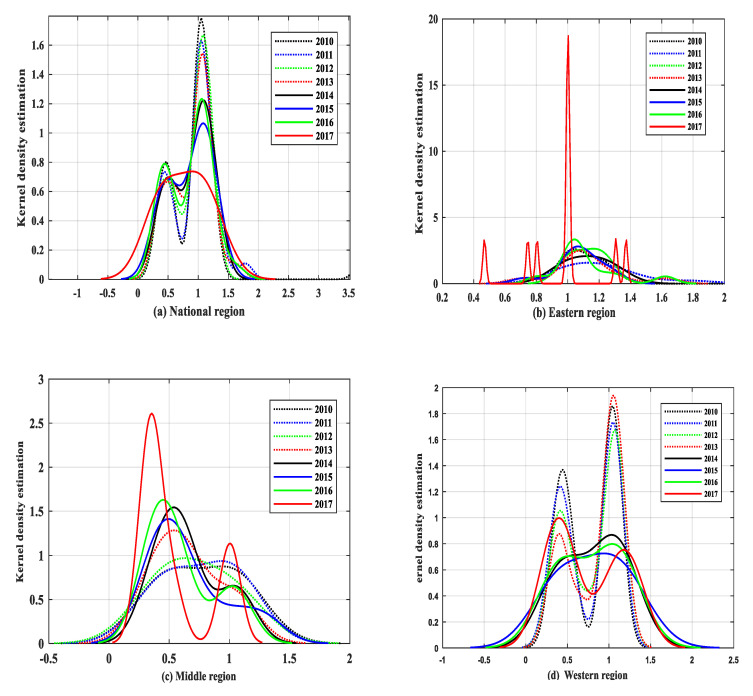
Kernel density curve of the (**a**) national region; (**b**) eastern region; (**c**) middle region; (**d**) western region (China).

Figure 4 summarizes the distribution of the kernel density curves in the national, east, middle, and western regions.

### 3.5. Climate to Agricultural Production and Crop Disaster Area

In terms of extreme weather days, most provinces have fewer than 200 days. The province with the lowest number of extreme weather is Yunnan, with 56.375 days. The province with the most extreme weather is Qinghai, with 230.375 days, accounting for about 63% of the year’s total. In terms of gross output value of agricultural, regional differences are also large, with a maximum difference of 4208.3625 billion CNY. Qinghai has the lowest total output value, which is 132.1875 billion CNY. The highest is Shandong at 4340.55 billion CNY. This is because extreme weather has a huge impact on agriculture.

There is a negative correlation between the gross output value of agricultural and extreme weather days. Qinghai, for example, has the highest number of extreme weather and the lowest gross output value for agriculture. The same is true of Beijing, which has relatively higher extreme weather days and a low agricultural output value (154.8625 billion CNY). In contrast, Sichuan has less extreme weather (95 days), and its gross output value of agricultural is high (3040.1625 billion CNY), which shows that extreme weather has a significant impact on agricultural economy. In turn, Shandong and Henan are special. Shandong, in particular, is China’s vegetable base, featuring a large shed for farming; its modern farming methods are also less vulnerable to extreme weather. 

**Figure 5:**
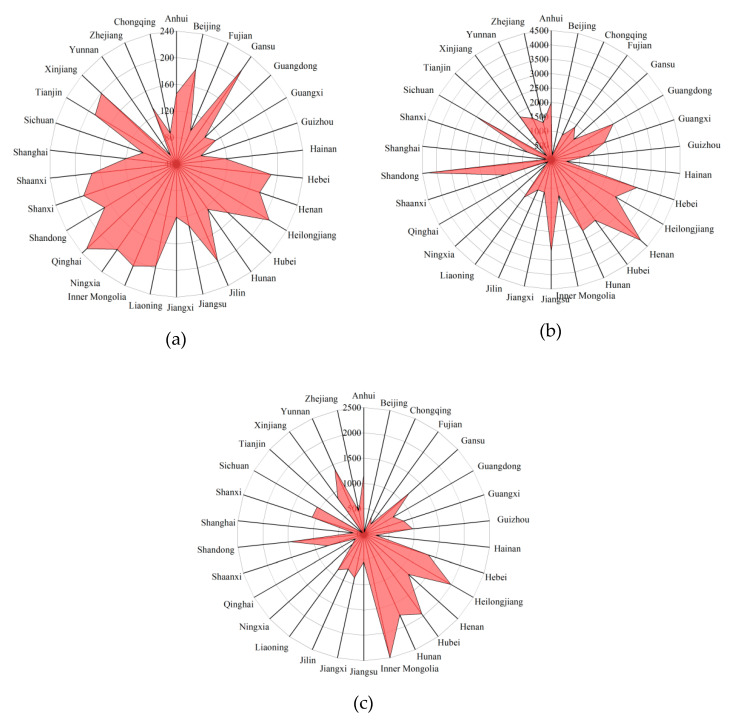
Radar maps of the following variables for each province over 8 years (China). (**a**) Extreme weather days (EWD); (**b**) gross output value of agriculture (GOVA); (**c**) crop disaster area (CDA).

Crop disaster area is positively correlated with extreme weather days. For example, there are more extreme weather in Inner Mongolia (198.75 days); its crop disaster area is 2498.675 thousand hectares, the largest in China. Zhejiang experiences less extreme weather (87.375 days) and its crop disaster area is smaller (469.4 thousand hectares). See Figure 5 for details.

**Figure 6:**
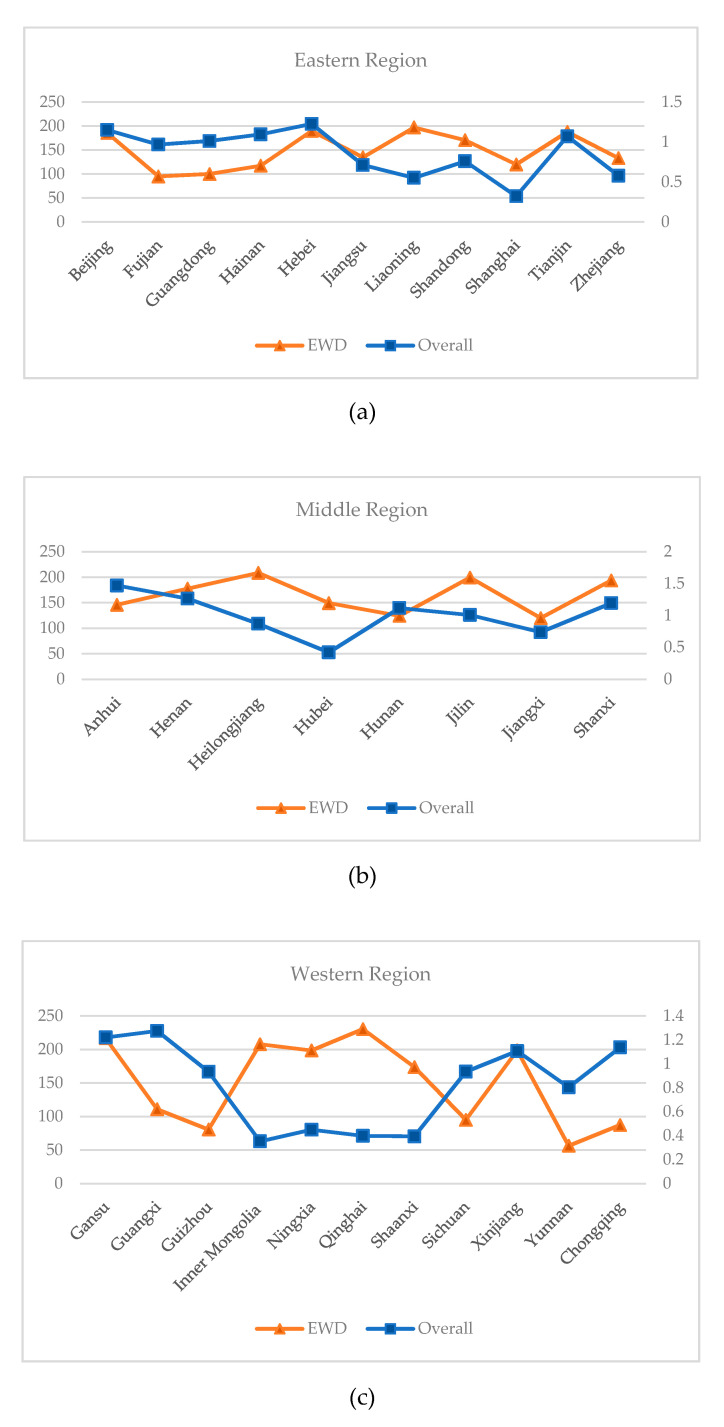
Line charts of EWD and overall agricultural production efficiency (China). (**a**) East; (**b**) Middle; (**c**) West.

In general, there is a negative correlation between extreme weather days and the overall agricultural production efficiency. As far as the eastern region is concerned, Fujian, Guangdong, Liaoning, and Shanghai show an obvious negative correlation. The more extreme the weather days, the less efficient the province’s agricultural production. In the middle region, the negative correlation is weak, but an inverse relationship can still be seen. In the western region, the negative correlation is obvious, especially in Inner Mongolia, Ningxia, Qinghai, and Shaanxi, with a large longitudinal wheelbase distance. It can be seen that extreme weather also has a huge impact on agricultural production. See Figure 6 for details.

### 3.6. Efficiency Analysis of Input-Output Index

In the input–output variables of agricultural production, the improvement value of GOVA is almost 0, and the improvement range is small. However, in some provinces, especially in the middle and western regions, the improvement values of AWC, CIA, and CDA are relatively large.

In the central region, the improvement values of Anhui and Jiangxi are higher, among which the CIA improvement value of Jiangxi is 1113.739, with a larger improvement range. Secondly, the improvement value of CDA in Hubei, Jilin, Jiangxi, and Shanxi is more than 500, and the highest improvement value is 968.968 in Hubei. In the western region, the improvement values of AWC, CIA, and CDA are higher in Inner Mongolia and Ningxia, and the highest CDA value is 2059.016 in Inner Mongolia. In addition, the average CIA improvement value in Inner Mongolia is 2043.147, with a large improvement range.

To sum up, among the input–output variables in China, the improvement range of GOVA in each region is relatively small, and each region maintains a high level of output. The improvement value of the CDA in input–output variables is the largest, which needs to be improved. Table 6 calculates the improvement values of the input–output variables in the abovementioned provinces over the past eight years. See Table 6 for details.

## 4. Conclusions

Based on the super-efficiency SBM model, Dagum Gini coefficient, and kernel density estimation method, this research analyzed the agricultural production efficiency and spatial differences of 30 provinces in China, obtaining the following conclusions and suggestions.

(1) Agricultural production efficiency is unevenly distributed. Among the three regions in China, the agricultural production efficiency of the eastern region is the best, and the agricultural production efficiency of the western region is inferior to other regions. As the eastern region is located in the coastal areas, its climate conditions and agricultural investment environment are better. With the eastern route of the south-to-north water diversion project entering a stable operation period, the water shortage in the north of China (Beijing, Tianjin, and Hebei) has been alleviated, and the agricultural production efficiency level in the north of China has remained above 1. In 2017, the precipitation in Liaoning reduced by 22%, and disasters such as drought, heavy rain, and strong wind occurred. Therefore, due to the impact of natural disasters, the agricultural production efficiency in Liaoning has declined significantly.

However, the middle region is located in the interior of China, dominated by a temperate, continental climate; so, its agricultural production efficiency level is low. The agricultural production efficiency decreases from 0.764 to 0.536. In the process of agricultural input and output, the improvement values of CIA and CDA in the middle region are high. It can be seen that the efficiency value of the above two variables should be increased in this region to promote the overall efficiency level. In the middle region, Henan and Heilongjiang, China’s major traditional agricultural provinces, have the right terrain and environment for crop production. Henan provides investment for agriculture through agricultural risk subsidy projects, which improves and optimizes the agricultural production efficiency of Henan. Heilongjiang, on the other hand, has a wide range of black land resources and water resources, so it has made great efforts to develop agricultural resources and improve its agricultural production efficiency by taking advantage of the centralized production of state-owned farms. In addition, the agricultural labor force population in Anhui has been lost due to the “siphoning effect” in Shanghai, Jiangsu, and Zhejiang, thus promoting the loss of agricultural production efficiency. It can be seen that the ladder efficiency gap in the middle region is relatively significant. The climatic features of the western region are mainly drought and little rain, and the agricultural production conditions are relatively harsh, but agricultural production efficiency fluctuates around 0.75. In the western region, Sichuan, Chongqing, Guangxi, and other provinces have abundant agricultural water, and a higher agricultural production efficiency has been achieved through manual development and mechanical operation. However, Gansu, Ningxia, and Inner Mongolia, due to their climatic features and topographical environment, have kept their agricultural production efficiency at a low level and failed to operate efficiently. Among them, the improvement values of the CDA in these three provinces are high, so the efficiency level of the variable should be effectively improved.

(2) Regional differentiation of agricultural production efficiency is significant. Based on the study of the Dagum Gini coefficient, it can be seen that the intra-regional Gini coefficient in the middle region and the western region is more differentiated, which is consistent with the above exploration of agricultural production efficiency. Under the intervention of the intra-regional environment, climatic differences, and economic factors, the intra-regional differences between the middle and western regions are larger than those between the eastern regions, and there is a ladder efficiency gap within the regions. As for the inter-regional Gini coefficient, it can be seen that the efficiency between regions fluctuates around 0.2. Due to the frequent population mobility and agricultural investment resources in the eastern and middle regions, which mainly flow from the middle region to the eastern region, the regional gap of the east–middle regions has increased significantly. The analysis of agricultural production efficiency between regions shows that the difference between the regions is the main source of regional development inequality. By using the kernel density estimation method, it is found that the concentration degree of the agricultural areas in the country decreases, the distribution of agricultural water resources in the regions is more balanced, and the differences between the different regions in the country gradually decrease. However, from the perspective of various regions, the integration of agricultural water resources in the eastern region is accelerating, and the distribution of agricultural resources tends to be concentrated. In 2017, the nuclear density estimation curve in the eastern region showed multiple peaks and the peak value increased. In the middle region, the right tail of the kernel density estimation curve moved slightly to the left, and the agricultural production efficiency showed a downward trend.

(3) All regions should strengthen the prevention and control of meteorological disasters. In recent years, as the global climate continues to warm, extreme weather events between regions have become more frequent, causing increasing losses and impacts. Among them, northeast China is most affected by low temperatures in winter, while hot weather in summer prevails throughout the country. In the face of severe high temperatures, local governments should organize the formulation of emergency plans. Under the premise of ensuring daily cultivation, local governments should reasonably improve the agricultural structure and layout and reform the farming system. By measuring the range of temperature changes in local areas, reasonable crops and water consumption can be used to improve the water-use efficiency. In addition, the relationship between crop growth and development, yield formation, and climatic conditions should be clarified in the reform of planting systems, so as to rationally utilize agro-climatic resources, prevent agro-climatic disasters, and improve the ability to cope with climate change. In the face of the low temperature disaster in some agricultural areas, each region should promote the improvement of crop insulation measures, by spraying warm water measures, supplement crop water, inhibit the evaporation of frozen tissue water, and promote tissue water absorption. Under the guarantee of early warning measures, farmers can build greenhouses and control light, temperature, and humidity in the sheds.

(4) All regions should ensure the supply of water for agriculture. As most of China’s agricultural areas are located in rural and suburban areas, their investment in facilities and the penetration rate of agricultural application technology are worse than those in urban areas. In the eastern region, the eastern route of the south-to-north water diversion project alleviates the disadvantages caused by the uneven distribution of water resources in the region, but it does not solve the “last kilometer” problem of agricultural irrigation. According to the distribution of local farmland and surrounding waters, the local township government and the county government should strengthen the construction of the last-stage water transmission channel, so as to make the south-to-north water diversion project truly benefit the majority of farmers to get rid of the vicious circle of agricultural water caused by long-term over-extraction of groundwater. However, the middle and western regions that the Yellow River flows through, such as Shaanxi, Ningxia, and Gansu, despite its rich resources for irrigation, produced “the river” problem because of the sediment deposition in the Yellow River, a faltering tributary, and the downstream riverbed elevation in the lower provinces. Furthermore, during the summer rainstorm, there is the danger of bursting the dike. Based on this situation, the provinces and regions in the Yellow River basin should set up unified and efficient regional remediation departments, with the support and guidance of the middle government. Based on the overall situation of the Yellow River basin, the governance problem of the Yellow River basin is planned. The safe supply of irrigation water and the smooth development of agricultural production in the agricultural areas of the river basin can be guaranteed by carrying out comprehensive control of soil and water conservation, “curve cut-off”, and other measures. In addition, according to the unified planning and arrangement of the Yellow River basin, the differences between regions and provinces should be weakened, and the differences in inter-region agricultural water use should be reduced through coordinated development. Through the above measures, China would be able to promote development within the region and reduce the differences between regions and within regions.

(5) All regions should promote the spread of water-saving measures in agriculture. In China, the main target of agricultural water-saving technology popularization is the farmers. However, the popularization of water-saving technology in China has been hindered to some extent due to the low educational level of agricultural employees, the deep-rooted thinking of traditional farming methods, and the poor operation and acceptance ability of agricultural water technology. In this context, village committees in different regions of China should make full use of agricultural assistance funds, guide local government departments to provide capital assistance for asset investment, and promote the popularization of water-saving agricultural facilities in rural areas and other remote areas. Moreover, advanced farmers should take the lead to improve the thinking of agricultural workers and reduce the obstacles to the popularization of water-saving technologies in agriculture. The coordinated planning and development of certain measures, such as the improvement of crop varieties, the overall arrangement of irrigation management modes, the change in thoughts about agricultural employees, and the use of the “spillover effect” to promote the spillover of agricultural water-saving technologies, would be conducive to the improvement of regional and inter-regional differences and thus promote a balanced development of agricultural water-use areas in China. These measures can effectively reduce the regional agricultural water concentration and promote the balanced development of the three regions in China.

## Figures and Tables

**Table 1:** Regional division of China.

Region	Provinces (Autonomous Regions and Municipalities)
East	Beijing, Tianjin, Shanghai, Liaoning, Hebei, Shandong, Jiangsu, Zhejiang, Fujian, Guangdong, Hainan
Middle	Heilongjiang, Jilin, Henan, Shanxi, Anhui, Hubei, Hunan, Jiangxi
West	Gansu, Guizhou, Ningxia, Qinghai, Shaanxi, Yunnan, Xinjiang, Sichuan, Guangxi, Chongqing, Inner Mongolia

**Table 2:** The input and output variables.

Stage		Variable	Unit
Agricultural production	Input	Agricultural Water Consumption (AWC)	million cubic meters
		Agricultural Employees (AE)	10,000 people
		Cultivated Irrigated Area (CIA)	1000 hectares
		Fixed Assets (FA)	100 million CNY
	Output	Gross Output Value of Agriculture (GOVA)	100 million CNY
		Crop Disaster Area (CDA)	1000 hectares
Climate variables		Extreme Weather Days (EWD)	day

**Table 3:** Descriptive statistics of the inputs and outputs.

Region	Variables	Mean	Median	Max	Min	SD
Nation	AWC	126.200	98.200	561.700	5.100	107.240
	AE	929.200	783.010	2712.000	37.090	653.090
	CIA	2133.050	1631.300	6031.000	115.500	1601.800
	GOVA	1694.390	1442.400	4929.900	92.100	1179.740
	CDA	909.790	734.300	4223.700	3.100	784.790
	EWD	153.460	158.000	240.000	42.000	50.350
	FA	2454.170	1983.300	10,663.630	93.450	1926.860
East	AWC	103.676	91.000	307.600	5.100	88.209
	AE	733.044	616.915	2273.100	37.090	642.804
	CIA	1834.947	1438.450	5191.100	115.500	1729.697
	GOVA	1687.623	1464.600	4929.900	129.800	1403.601
	CDA	555.800	405.200	2582.300	3.100	589.529
	EWD	148.159	149.000	219.000	74.000	41.016
	FA	2099.322	1336.964	8782.641	93.451	2098.056
Middle	AWC	152.210	154.950	316.400	37.980	71.885
	AE	1251.662	1083.300	2712.000	491.400	659.540
	CIA	3148.032	2877.200	6031.000	1274.150	1437.446
	GOVA	2153.923	2089.400	4610.700	669.000	1098.482
	CDA	1343.639	1174.400	4223.700	225.100	767.538
	EWD	164.750	168.500	224.000	87.000	38.431
	FA	3426.860	2981.287	10,663.634	1086.916	1934.765
West	AWC	129.816	94.900	561.700	19.200	138.005
	AE	890.826	790.500	2083.200	114.790	571.502
	CIA	1692.973	1297.350	4982.000	182.500	1231.581
	GOVA	1366.955	1333.850	4004.200	92.100	846.001
	CDA	948.256	753.400	3917.300	70.800	806.888
	EWD	150.557	169.500	240.000	42.000	63.897
	FA	2101.599	1835.162	6343.983	336.971	1462.148

**Table 4:** Overall efficiency by provinces from 2010 to 2017 (China).

Region	DMU	Overall	2010	2011	2012	2013	2014	2015	2016	2017
East	Shanghai	1.200	1.291	1.321	1.276	1.087	1.118	1.091	1.622	1.000
	Shandong	1.093	1.082	1.096	1.061	1.106	1.229	1.099	1.084	1.011
	Tianjin	1.218	1.207	1.781	1.144	1.628	1.201	1.086	1.068	1.000
	Beijing	1.249	3.706	1.285	1.209	1.205	1.087	1.199	1.057	1.000
	Jiangsu	1.272	1.198	1.214	1.231	1.332	1.291	1.304	1.317	1.307
	Hebei	1.012	1.000	1.016	1.041	1.037	1.000	1.000	1.000	1.000
	Hainan	1.217	1.190	1.190	1.200	1.163	1.198	1.233	1.214	1.372
	Zhejiang	0.857	1.000	1.000	1.000	0.707	0.912	0.731	0.806	0.804
	Fujian	1.137	1.069	1.373	1.184	1.093	1.342	1.071	1.072	1.000
	Guangdong	0.967	1.000	1.000	1.000	1.000	1.000	1.000	1.000	0.745
	Liaoning	0.910	1.000	1.000	1.000	1.000	1.000	1.000	1.000	0.469
Middle	Shanxi	0.448	0.527	0.536	0.507	0.466	0.468	0.407	0.376	0.310
	Jilin	0.546	0.579	0.621	0.620	0.681	0.593	0.562	0.466	0.292
	Anhui	0.421	0.477	0.447	0.443	0.418	0.458	0.395	0.365	0.366
	Jiangxi	0.353	0.338	0.344	0.347	0.312	0.354	0.378	0.380	0.368
	Henan	1.110	1.188	1.109	1.107	1.075	1.089	1.288	1.064	1.010
	Hubei	0.690	1.000	1.000	0.686	0.649	0.656	0.577	0.583	0.541
	Hunan	0.707	1.000	1.000	1.000	0.596	0.632	0.675	0.592	0.403
	Heilongjiang	1.007	1.000	1.000	1.000	1.031	1.022	1.000	1.000	1.000
West	Yunnan	0.563	0.463	0.420	0.518	1.000	0.651	0.556	0.515	0.503
	Sichuan	1.104	1.110	1.052	1.083	1.044	1.091	1.096	1.127	1.248
	Gansu	0.397	0.462	0.406	0.411	0.421	0.400	0.384	0.410	0.295
	Ningxia	0.321	0.336	0.300	0.327	0.314	0.310	0.321	0.341	0.323
	Qinghai	0.893	1.000	1.000	1.000	1.000	1.000	1.000	1.000	0.474
	Chongqing	1.137	1.108	1.108	1.108	1.117	1.113	1.172	1.127	1.243
	Xinjiang	0.776	1.000	1.000	1.000	1.000	1.000	0.478	0.506	0.448
	Inner Mongolia	0.392	0.398	0.429	0.405	0.433	0.408	0.388	0.349	0.338
	Guangxi	0.741	0.561	0.583	0.731	0.677	0.523	1.000	1.000	1.000
	Guizhou	1.181	1.032	1.022	1.123	1.172	1.264	1.339	1.281	1.308
	Shaanxi	1.070	1.022	1.119	1.102	1.085	1.083	1.055	1.050	1.047

**Table 5:** The Dagum Gini coefficient and its decomposition results.

Year	Overall	Intra-Regional Difference			Inter-Regional Difference			Contribution Rates (%)		
		East	Middle	West	East–Middle	East–West	Middle–West	G_w_	G_nb_	G_t_
2010	0.2445	0.1952	0.2130	0.2136	0.2847	0.2786	0.2170	28.81%	55.87%	15.32%
2011	0.2040	0.0960	0.2042	0.2240	0.2340	0.2325	0.2181	26.31%	55.11%	18.58%
2012	0.1810	0.0497	0.2113	0.2119	0.2279	0.1816	0.2238	25.32%	56.59%	18.10%
2013	0.1978	0.1004	0.2176	0.1917	0.2765	0.1758	0.2430	26.54%	57.74%	15.72%
2014	0.2001	0.0663	0.2032	0.2290	0.2692	0.1943	0.2450	25.07%	58.26%	16.67%
2015	0.2124	0.0710	0.2417	0.2475	0.2732	0.1992	0.2706	26.29%	49.67%	24.04%
2016	0.2227	0.0917	0.2270	0.2410	0.3055	0.2110	0.2706	25.67%	58.24%	16.09%
2017	0.2604	0.1254	0.2634	0.2919	0.3216	0.2513	0.3195	27.69%	46.53%	25.77%

Notes: G_w_ refers to intra-regional differences; Gnb refers to inter-regional differences; Gt refers to intensity of transvariation.

**Table 6:** Improvement value of the input–output variables.

Region	DMU	AWC (MCM)	AE (10^4^ P)	CIA (10^3^ H)	GOVA (10^8^ CNY)	CDA (10^3^ H)
East	Beijing	0.000	0.000	0.000	0.000	0.000
	Fujian	0.000	0.000	0.000	0.000	0.000
	Guangdong	15.365	30.405	0.000	0.000	1.947
	Hainan	0.000	0.000	0.000	0.000	0.000
	Hebei	0.000	0.000	0.000	0.000	0.000
	Jiangsu	0.000	0.000	0.000	0.000	0.000
	Liaoning	4.839	9.474	84.451	0.000	100.238
	Shandong	0.000	0.000	0.000	0.000	0.000
	Shanghai	0.000	0.000	0.000	0.000	0.000
	Tianjin	0.000	0.000	0.000	0.000	0.000
	Zhejiang	0.179	0.000	119.610	0.000	173.353
Middle	Anhui	59.818	488.697	2456.613	0.000	808.082
	Henan	0.000	0.000	0.000	0.000	0.000
	Heilongjiang	0.000	0.000	0.000	0.000	0.000
	Hubei	16.606	171.319	428.170	0.000	968.968
	Hunan	34.446	223.382	354.586	0.000	675.631
	Jilin	28.329	21.497	770.171	0.000	524.753
	Jiangxi	108.350	287.984	1113.739	0.000	598.426
	Shanxi	13.498	240.398	687.358	1.160	799.944
West	Gansu	40.920	415.942	636.620	0.000	826.647
	Guangxi	60.035	335.971	11.894	0.000	297.941
	Guizhou	0.000	0.000	0.000	0.000	0.000
	Inner Mongolia	91.868	11.348	2043.147	0.000	2059.016
	Ningxia	44.304	66.551	250.166	0.616	234.231
	Qinghai	0.582	2.986	0.000	12.636	32.681
	Shaanxi	0.000	0.000	0.000	0.000	0.000
	Sichuan	0.000	0.000	0.000	0.000	0.000
	Xinjiang	141.622	0.000	950.458	0.000	108.955
	Yunnan	28.447	610.461	252.999	15.049	844.679
	Chongqing	0.000	0.000	0.000	0.000	0.000
